# Complementation Studies of Bacteriophage λ O Amber Mutants by Allelic Forms of O Expressed from Plasmid, and O-P Interaction Phenotypes

**DOI:** 10.3390/antibiotics7020031

**Published:** 2018-04-05

**Authors:** Sidney Hayes, Karthic Rajamanickam, Connie Hayes

**Affiliations:** Department of Microbiology and Immunology, College of Medicine, University of Saskatchewan, Saskatoon, SK S7N 5E5, Canada; kar029@mail.usask.ca (K.R.); clh127@outlook.com (C.H.)

**Keywords:** bacteriophage lambda (λ), bi-directional replication initiation from *ori*λ, O and P initiator proteins, *ori*λ interaction site, O complementation by *ori*λ-defective alleles, influence of O:P interactions on cell growth and O activity

## Abstract

λ genes O and P are required for replication initiation from the bacteriophage λ origin site, *ori*λ, located within gene *O*. Questions have persisted for years about whether O-defects can indeed be complemented *in trans*. We show the effect of original null mutations in O and the influence of four origin mutations (three are in-frame deletions and one is a point mutation) on complementation. This is the first demonstration that O proteins with internal deletions can complement for O activity, and that expression of the N-terminal portion of gene P can completely prevent O complementation. We show that *O-P* co-expression can limit the lethal effect of P on cell growth. We explore the influence of the contiguous small RNA OOP on O complementation and P-lethality.

## 1. Introduction

**Figure 1:**
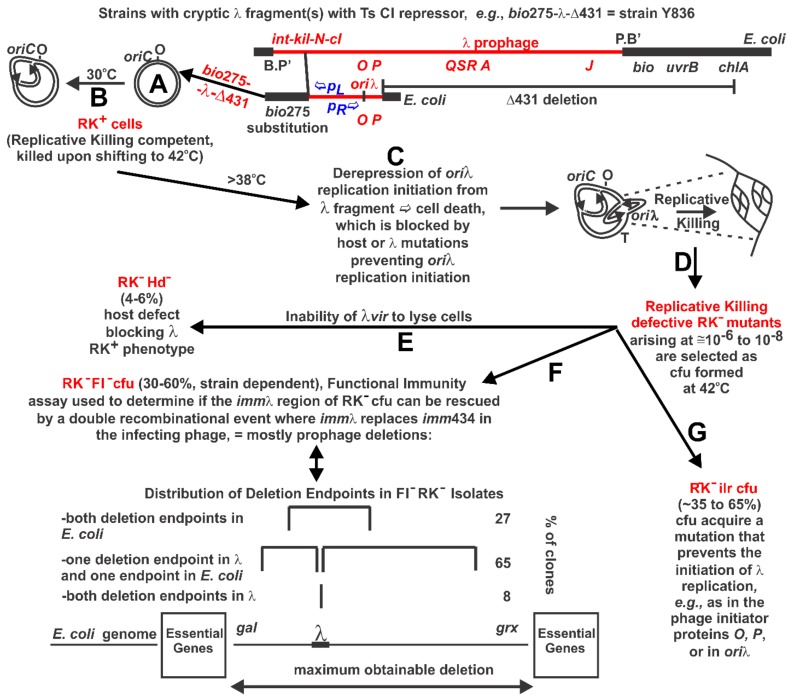
Replicative Killing, RK^+^ phenotype and selection for RK^−^ mutants. (**A**) Defective prophage strains were made where the *int-kil* or *int-ral* genes of λ were substituted with the *bio*275 or *bio*10 regions of specialized transducing phage to remove a phenotype termed “killing to the left”, dependent on *kil* [23]. The starting cells included the *chlA* deletion Δ434 that removed all of the late genes, i.e., cell lysis, head and tail for λ [11]. These constructs include (i) an active *imm*λ region with gene *cI*[Ts]857 encoding a repressor that blocks transcription from promoters *pL* and *pR* along with the *cro* repressor just right of *pR*, and (ii) the *rep*λ region that includes genes *O* and *P* and the *ori*λ target for replication initiation from the λ genome. The genome for strain Y836, shown, has the *bio*^+^ operon to the left of the λ fragment and Δ431deletion to the right; (**B**) As long as strain Y836 maintains CI repressor activity the cells can grow normally without gene expression from the repressed λ fragment; (**C**) When the cells are shifted to growth conditions where the CI[Ts] repressor loses its ability to block transcription from *pL* and *pR* the remaining λ genes become derepressed, the phage replication initiation genes *O* and *P* are expressed and rounds of replication initiation arise from *ori*λ. The λ replication forks extend bidirectionally into the adjacent regions of the *E. coli* genome, likely colliding with *E. coli* replication forks. The event is highly lethal to the cell because the λ fragment has no mechanism for excision from the genome and was termed Replicative Killing [18]; (**D**) When cells with a conditionally repressible defective λ prophage are shifted from growth at 30 °C to 42 °C the Replicative Killing, RK^+^, phenotype is triggered, resulting in cell death. Rare mutations that suppress the loss of λ replication control are selected as RK^−^ clones capable of colony formation at 42 °C. These survivor CFU have lost the capacity for λ replication. This strategy is based on the PDS selection [15], where an intact prophage is made *N*-defective, so that expression of *int-xis* and late/cell lysis gene expression is limited without *N*-antitermination of *pL* and *pR* transcription upon prophage induction. There are many possibilities for RK^−^ mutants; (**E**) Cells acquiring defects in host genes participating λ replication are termed RK^−^ Hd^−^. For example, the GrpD55 mutation in *dnaB* is of this type, though not isolated as shown [24,25]; (**F**) A marker rescue recombination assay is used to determine if the *imm*λ regions genes and target sites remain functional (i.e., FI^+^) when substituted for the *imm*434 region of a hybrid phage. The FI assay scores for the activity of the *pR* promoter, but in practice it is a good indication of whether the λ fragment in Y836 cells was partially or fully deleted. An example of the deletion endpoints of RK^−^ FI^−^ mutants from Y836 is shown [26,27,28]; (**G**) It was found that brief pretreatment RK^+^ of cells held at 30 °C with a mutagenic substance, prior to shifting them to 42 °C increases the frequency of RK^−^ mutants. This assay, termed the RK Mutatest, proved very sensitive due to the rather large target potential for RK^−^ mutants [29,30,31].

Bacteriophage λ prophage is maintained within the chromosome of *Escherichia coli* cells by its CI repressor protein, which prevents the transcription of λ genes positioned leftward and rightward from promoters *pL* and *pR* that straddle *cI* (Figure 1A). CI binds to operator sites that overlap these promoters. Upon inactivation of CI, the derepressed prophage genes *N*-*int* are expressed from *pL*, and genes *cro-cII-O-P-Q* are expressed from *pR*. Transcription initiated from *pR* requires gpN activity to proceed effectively past the rho-dependent *t_R_*_1_ termination site positioned between *cro* and *cII* (reviewed in [1,2,3,4]).

The mechanism for bi-directional initiation of λ DNA replication involves a complex interaction of phage proteins gpO and gpP (designated herein as O and P) with *E. coli* host DNA replication proteins. In brief, O acts to bind the replicator site, *ori*λ, or origin for replication initiation that is situated midway within the *O* sequence [5,6]. The P protein recruits DnaB, the major replicative helicase for unwinding double-stranded DNA, bringing it to *ori*λ-bound O to form a DnaB:P:O:*ori*λ preprimosomal complex. P can commandeer DnaB away from its cellular equivalent, DnaC [7]. Since the interaction of P with DnaB inactivates the helicase activity of DnaB, the dissociation of P bound to DnaB in the preprimosomal complex is required to restore DnaB activity, which involves *E. coli* heat shock proteins DnaK, DnaJ, and GrpE [8,9]. By inhibiting transcription from *pR*, CI blocks a *cis* requirement for replication initiation described as transcriptional activation, explaining why providing O and P from a superinfecting heteroimmune phage will not stimulate replication initiation from an integrated resident *imm*λ prophage [10,11]. This requirement for *pR* transcription can be suppressed by *ri*^C^ (replicative-inhibition constitutive) mutations which lie outside of *ori*λ [12,13].

The excision of a λ prophage from the host chromosome between B.P’ and P.B’ sites (Figure 1A) is dependent upon λ genes *int* and *xis* (reviewed in [1,4,14]. Their expression requires that gpN antiterminate transcription at *t_L_* terminator signals positioned between *pL-N* and ahead of *xis*. The PDS selection (refer to Abbreviations, Unique λ Terminology) for cell survivors of λ *N cI* ([Ts], temperature sensitive) prophage, named for its inventor [15], takes advantage of the induced prophage being unable to excise from the chromosome or lyse its host cell. In addition, the *N* null mutation reduces late λ gene expression and cell lysis, which depend on N for transcriptional antitermination at several *t_R_* sites upstream of the late genes. Examinations for *E. coli* cell survival using the PDS selection led to the suggestion that mutations preventing the initiation of λ replication suppress cell killing [15,16,17,18,19,20,21].

The induction lethality phenotype for non-excisable prophage was termed Replicative Killing [18]. Accordingly, starting cells with de-repressible, but non-excisable prophage, as in Figure 1A and B, are termed RK^+^ (Replicative Killing competent) cells and the selected survivor cells that form CFU at 42 °C were named Replicative Killing defective (RK^−^) mutants (Figure 1C–G). The concept evolved that the starting cells possess the capacity for λ replication initiation upon prophage induction, whereas the survivor clones do not. The results of these early studies were reviewed [22]. Only a few mutations conferring the RK^−^ phenotype for survivor CFU derived from the PDS selection or those from the N^+^ λ fragment strains (an example is shown in Figure 1) have been characterized by DNA sequence analysis. Most all the RK^−^ mutants were obtained before the possibility for PCR amplification of a mutated region of the chromosome, which enables direct sequence determination of the RK^−^ mutation. Hence, those RK^−^ mutations that have been characterized depended mainly upon genetic analysis using phage mapping and complementation.

Genetic mapping of RK^−^ mutations within *O* or *P* requires that both *O* and *P* initiator gene products can complement and function *in trans*. When a cell is infected with two phages, one defective in *O* and the other in *P*, complementation is observed suggesting that the products of these genes are diffusible [32]. However, Rao and Rodgers [33] were unable to demonstrate that cells carrying a ColE1 plasmid expressing *O*^+^ could complement, i.e., support the efficient plating of an infecting *λimm*21 *O*am29 phage, even though the plasmid copy number varied between 50 copies at 32 °C and 260 copies at 42 °C. However, they could demonstrate *trans* complementation for phages with amber mutations in *N* or *P* by plasmids that can express these genes. Kleckner [34] suggested that *O* might act *in cis* or be poorly complemented under *N* defective conditions, and other experiments suggesting that O functions in *cis* were reported in [22]. These findings throw into question whether it is possible to designate using a phage complementation assay whether an induced RK^−^ mutant has an *O*^+^ or *O*^−^ phenotype. Alternatively, these divergent observations suggest that the ability of *O* to complement is more complex than initially assumed. A complicating problem in addressing this historical issue is that the mutant λ phages used in these early complementation assays were never subjected to DNA sequence analysis, so that in many cases their designations depend only upon unreported phage mapping studies, without accompanying proof of mutational site determination.

In this report, we examine the sequences of some early *O* mutations provided by A. Campbell from his original collection [35], and phages we have acquired over the years from laboratories that have participated in studies on *O*. We have cloned out *O* alleles from the chromosomes of RK^−^ mutants and inserted them into a plasmid where the expression of the allele is regulated by CI[Ts], and is repressed in cells growing at 30 °C, or can be slightly to fully induced at growth temperatures between 37 °C to 42 °C. Each of these alleles was examined for their ability to complement the growth of a λ *O* amber mutant(s) *in trans*. We have explored the influence of O:P interactions on cell growth and toxicity, since the expression of *P* by itself, using the same system, is highly toxic [25,36]. These studies reveal that some alleles of *O* with internal deletions can complement as well or better than *O*^+^ and that the co-expression of *O-P*, or of *O* with portions of the N-terminal end of *P*, prevents an ability of *O* to complement *in trans*.

## 2. Results

### 2.1. Taking Stock of O Mutations in Phage and Prophage Collections

**Figure 2:**
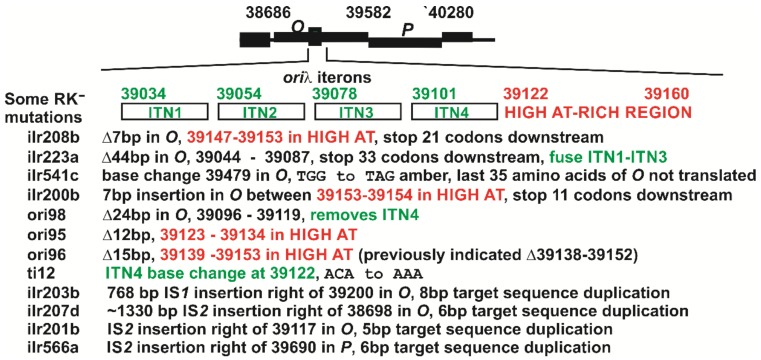
RK^−^ ilr mutations characterized within λ genes *O-P*. The minimal *ori*λ size was suggested to include a HIGH-AT-rich region to the right of four iteron sequences, ITN’s1-4 [43,44], which each contain an 18 bp inverted repeat of hyphenated symmetry, joined by adenine residues that can cause *ori*λ to assume a bent structure [45]. The mutants shown designated ori95, ori96 and ori98 were obtained from WD from prophage with *r*-mutants *r-95*, *r-96*, and *r-98*. Note that Denniston-Thompson, et al., [46] sequenced the *r*-mutants *r-99*, *r-96* and *r93* which represent Δ12 bp (39120–39131), Δ15 bp (39138–39152) and Δ24 bp (39092–39115) [42]. Our sequence localization for ori96 (*r96*) differs by one bp from that assigned in [42].

DNA sequence characterization of *O* mutations in phage and prophage collections available to us is summarized, Table 1. Campbell (AC) described and mapped *O*am mutations 8, 29 and 125 [35]. Furth genetically mapped *O*am mutations and ordered them (N- to C-terminal) 905, 29, 1005, 8, 125, 205 by marker rescue using six prophage strains, each with a deletion designated as extending into *O* [37,40]. The original AC prophage in strain R573 representing *O*am125 included two missense mutations in addition to an amber mutation at 39511 bpλ. These three mutations were carried on a phage (our lysate #1024, Table 1) from LT designated MMS254. In contrast, isolate #1023 for *O*am29 designated LT-MMS99 included a silent mutation in addition to the amber mutation at 39511. Of relevance, four phage lysates (designated as carrying *O*am8 or *O*am29 mutations) that were obtained from researchers were found WT for λ genes *cII-O-P* through base 40712 in orf *ninB*, but they did include a nonsense mutation somewhere in λ since they grew well on a *supE* host but not on a *sup*^o^ host. These results can explain why the initial assignment of an *O*^+^ phenotype to some RK^−^ ilr mutants proved incorrect (Figure 2).

### 2.2. Replicative Killing Selection and Mutants

The selection of RK^−^ mutants with defects in λ replication initiation were categorized (Figure 1) as follows: RK^−^ clones designated Hd^−^ (host defects), representing about 4% to 6% of selected spontaneous RK^−^ clones [22], are not lysed by λ*vir*. These mutants are arbitrarily considered to have an altered host gene whose product participates in vegetative λ growth, or a λ-fragment mutation whose effect is to complement negatively for the growth of λ*vir*. The RK^−^ clones that are lysed by λ*vir*, retain the *imm*λ phenotype at 30 °C, and were FI^+^, were designated RK^−^ ilr (initiation of λ replication defective). The FI, or functional immunity assay [11,41], represents a stab of an RK^−^ CFU to a lawn of cells lysogenized with λ*imm*434T to which is added free λ*imm*434*cI*. If *imm*λ double recombinant phage can be generated (indicated by a lysis area forming around the RK^−^ clone stabbed to the overlay plate), this is taken to indicate that the *imm*λ region encoding *o*L/*p*L -*cI*- *o*_R_/*p*R is functional both in the RK^−^ mutant and in the *imm*λ recombinant. The RK^−^ FI^−^ Imm^−^ isolates mainly have had large deletions (>10 Kb) [26,27,28]. In an examination of the spontaneous RK^−^ mutants/mutations arising from four RK^+^ *N*^+^ selector strains, 256/650 RK^−^ isolates were the RK^−^ ilr type [22].

Figure 2 shows eight sequences for RK^−^ ilr mutants falling within *O-P* that were derived from induced *N*^+^ prophage, along with mutants derived from induced *N*^−^ prophage, including ori-95, -96, -98 obtained by Rambach [20] and ti12 from the Dove laboratory [18]). The sequences for ori95 and ori98 were not previously reported [42]. The ori96 mutation was a 15 bp deletion of λ bases 39139–39153 (not 39138–39152 as reported [42]). Mutation ori98 removed the entire iteron-ITN4 region, and ori95 and ori96 each deleted part of the High-AT rich region within *O*. Except for ilr541c, which included a stop codon that eliminated translation of the last 35 codons of *O*, the remaining RK^−^ ilr *O* mutations (Figure 2) represented small deletions within *O* or insertions that could exert a polar effect on downstream *P* expression. Each of the ilr mutants were initially scored as being *O*^+^, which clearly was not proved correct by sequence analysis. None of the ilr mutations had in-frame deletions within *O* as with those obtained in Rambach’s λ *N*^−^ selection.

### 2.3. Complementation for O Activity in Trans

**Figure 3:**
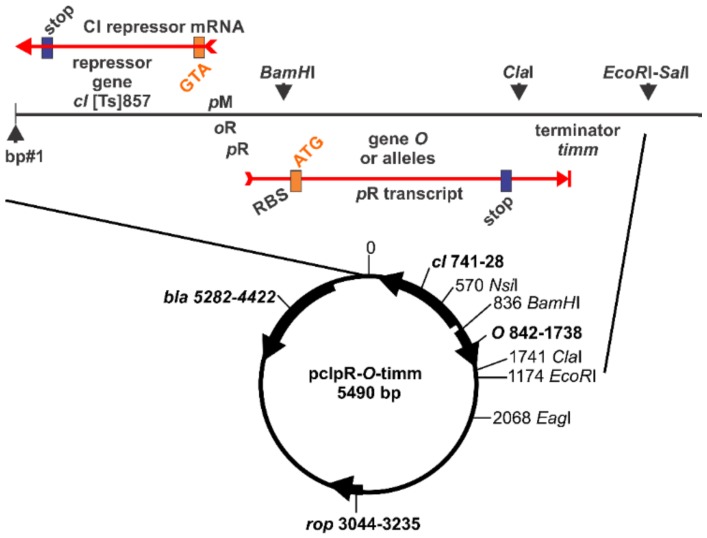
Expression vector pcIpR-(*O* alleles)-timm. Expression of gene *O* or an allele occurs upon inactivation of the CI repressor by raising cells grown at 30 °C to 42 °C. Immediately following the 299 codons of *O* is an ochre stop codon, where the last base in TAA represents the first base of the *Cla*I restriction site ATCGAT.

The wild type *O* protein is 299 amino acids (AA) [43]. Alleles of *O* were cloned into an expression plasmid, Figure 3. Each allele was from an RK^−^ mutant for which complementation analysis had suggested was *O*^+^.

Each plasmid was transformed into 594 cells, creating strains as 594[pcIpR-*O*-timm] that were used as hosts for λ *O*am plating. The assays for *O* complementation were incubated at 30, 37, 39 and 42 °C. The results for plating assays at 42 °C, where the *O* allele is fully expressed, are shown in Table 2. Phages whose *O* allele produced 76 or 294 AA’s of O were weakly complemented by O^+^, whereas the *O*am905 mutation expressing 37 AA of O was not complemented by O^+^. Complementation was improved 5- to 6-fold in two of the three λ *O*am suppression assays by the addition of a SPA tag to the COOH end of the wild type *O* sequence, i.e., the addition of a seven AA linker (GGSGAPM) joined to the 69 AA SPA tag [47] sequence. The *O*-*ori*:98 mutation removing ITN4 within *O* was incapable of complementing for O. Remarkably, *O* alleles with the *ori*:ti12 point mutation in ITN4 and those with *ori*:95 and *ori*:96 in-frame deletions, respectively, of 12 and 15 bp’s within the High AT-rich region of *ori*λ, were each able to complement all three *O*am mutants. Any condition where P, or a portion of the N-terminal end of P was expressed, completely prevented O-complementation. Induced prophage strains with insertions within *P*, but with sequenced intact *O* genes that would be fully derepressed when shifted to 42 °C were incapable of providing for O complementation. The RK^−^ ilr mutants, previously designated phenotypically as *O*^+^ [11], proved to have insertions or deletions in *O* and should not complement, as was found for the cloned prophage *O* genes from mutants 208b, 223a and 541c (each of which could complement for *P* [36]).

### 2.4. Influence of O-P Co-Expression on Cell Growth, P-Lethality, and Plasmid Loss

**Figure 4:**
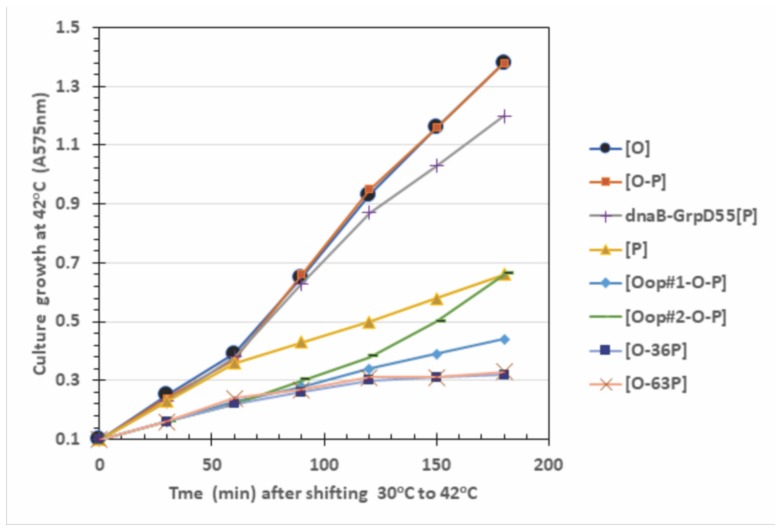
Influence of induced *O*, *P* gene expression on cell growth at 42 °C. All the strains were made by transforming hosts 594 or 594 *dnaB*-GrpD55 with pcIpR-(‥)-timm plasmids that included the cloned *O*, *P* DNA fragment, and selecting the transformants on LBAmp50 agar (medium composition is described in footnote to Table 3). The plasmid inserts in each CFU employed were verified by DNA sequence analysis. Cells were inoculated from overnight cultures grown up overnight in LBAmp50 broth and then 0.4 mL of culture was added to triplicate 20 mL fresh LB cultures that were incubated at 30 °C for ~30 min to reach an A575 = 0.1. Upon reaching an absorbance of 0.1 the cultures were transferred to a shaking 42 °C water bath. Aliquots were sampled every 30 min for 3 h. The average absorbance is shown, with a standard error for each culture time of less than 5% the averaged absorbance value.

The expression of P, or N-terminal fragments of P, block O complementation (Table 2). If O and P expression can influence O complementation, does their co-expression negate P-lethality? Figure 4 shows that *O* expression alone, or the co-expression of WT genes *O* and *P* over a span of four doublings in culture absorbance (with 45 min per doubling) did not perturb cell growth for cultures shifted from growth at 30 °C to 42 °C. In contrast, expressing *P* alone, or constructs expressing *oop*-*O-P*, or constructs that were WT for *O* but could express a portion of the N-terminal region of *P* were each highly inhibitory to cell growth. Table 3 shows the effect of *O-P* constructs on cell viability and plasmid loss. We re-examined several of the observations reported in [25], where the expression of *P*, even in trace levels at 37 °C, kills about 99% of the transformed cells and all plasmids were lost in survivor CFU’s. The lethality of P is completely suppressed by two missense mutations in *dnaB* that comprise the allele *dnaB*-GrpD55. Expressed by itself, O is not toxic and does not cause plasmid loss. Co-expression of *O-P* reduces the cellular toxicity of *P* expression alone by 12 to 15-fold at 37 and 39 °C. Co-expression of *oop-O-P* significantly prevents plasmid loss at 37 and 39 °C but exerts a minimal effect on cell viability. The inclusion of portions of the N-terminal end of *P* plus *O* significantly reduces cell viability and plasmid retention at 39 and 42 °C, compared to the expression of only *O*.

The *oop* DNA sequence encodes a 77-base noncoding small RNA that is transcribed in an antisense orientation to *O*. Part of the *oop* sequence overlaps the sequence for gene *cII* preceding *O*, with the *pO* promoter for *oop* transcription overlapping the N-terminal end of the *O* sequence. The #1 and #2 *oop*-*O* or *oop-O-P* constructs are deleted for the N-terminal end of cII. Since *O* or *O-P* are directly transcribed from *pR* on the plasmid, we asked if the antisense *oop* transcript would influence O or P activity expressed from the plasmid. Except for its ability to support some low-level suppression of λ*O*am905 plating, OOP RNA expression does not significantly influence O complementation (Table 2); however, it exhibits a profound effect on the ability of P expression to evoke plasmid loss (Table 3), over and above the quenching influence of *O-P* co-expression on cellular P-lethality. A hypothesis is that OOP RNA hybridization to the *pR-oop-O-P* mRNA expressed from the induced plasmid reduces downstream *P* translation/accumulation within the cell.

## 3. Discussion

### 3.1. O-Complementation

Our sequencing of *O*am29 reveals that this *O* allele encodes 294 of 299 amino acids. Thus, the last five amino acids of O are essential for O activity, and yet 76 amino acids, i.e., a linker and the SPA tag sequence, can be added to its COOH-terminal end, with the effect of improving the ability of O to complement.

Until this report, no one appears to have determined if in-frame deletions within *O* can influence its ability to complement, or will simply nullify its activity. We show that the 24 bp deletion in ori98 (*r98*) removing ITN4 nullifies the ability of O to complement; however, the 12 and 15 bp deletions in ori95 and ori96 (*r95*, r96), each falling within the High-AT rich region of *ori*λ improved the ability of O to complement. In addition, the *ori*λ *ti*12 mutation, representing a mismatch changing threonine to lysine within the ITN4 interval seemed to improve, rather than reduce, O complementation.

We were unable to demonstrate O complementation or saw extremely poor complementation for two sequenced O^+^ prophages in *N*^+^ RK^−^ cells, each of which had acquired insertions within *P*, i.e., strains ilr566a and Bib11t. This result leads us to question whether it is possible to demonstrate complementation where the prophage for the RK^−^ cells carries a *N*^−^ mutation and would poorly express *O*, e.g., Rambach’s conclusion that the *r*96 mutant isolated from an *N*^−^ prophage complemented for O. Indeed, full *O*^+^ expression from the pcIpR-*O*-timm plasmid in cells plated at 42 °C did not complement (i.e., support plaque formation of) a phage with an *O*am905 mutation.

### 3.2. O:P Interaction Effects

The functional cooperation of O and P in λ replication initiation was suggested by genetic studies [49]. The N-terminal region of O was suggested to contain a DNA binding domain and the COOH-terminal region to contain a P-binding domain [40,50,51], with the domains separated by a flexible linker region [52]. Tsurimoto and Matsubara [5] showed that O protein binds to each ITN as a dimer, thus *ori*λ should bind four dimers, with higher order binding suggested [53] to form an O-some that produces torsional stress on the adjacent AT rich region causing the double-stranded DNA to become slightly destabilized and partially unwound [54]. The N-terminal portion of P was assumed to contain an O-binding domain [55], while its COOH-terminal domain was suggested to interact with the host DnaB replicative helicase [55,56]. It has been suggested that a complex between O and P is formed that can be independent of DnaB [51,57].

In essence, the idea was advanced that O bound to *ori*λ is a display platform that is recognized by P:DnaB. However, we show that the co-expression of *O-P* results in several phenotypic effects which suggest that this idea is too simplistic. The co-expression of *O-P* nullifies the inhibitory effect of *P* expression on cell growth, for over four hours, and it reduces cell killing caused by prolonged expression of P (i.e., when expressed at 39 °C). In contrast, the co-expression of *O-P* nullifies the ability of O to complement. These opposed activities suggest that O and P physically interact without having O bound to *ori*λ. Combining O expression with the possibility for expression of the N-terminal portion of P eliminates O complementation, suggesting that O binding to the N-terminal portion of P prevents its useful binding to *ori*λ, which is presumably a requirement for O complementation activity. However, the co-expression *O-P* does not temper the ability of P to cause plasmid loss. Thus, while the expression of *P*, or N-terminal portions of *P*, can obviate O complementation, coordinate *O* expression does not fully nullify all the P-lethality phenotypes.

### 3.3. RK^−^ Mutant Selection Considerations

Dove and Blattner’s laboratories collaborated in mapping [40] and sequencing [46] some of the nine *r* mutants selected by Rambach [20], who based his selection on the assumption that the “replicator” gene was different from initiator genes *O* or *P*. They concluded that regions of the initiator gene, i.e., *O*, overlap the replicator site, now termed *ori*λ. We show herein that regions of *ori*λ are not essential for activity of the O initiator protein. We previously demonstrated [39] that λ *N*am7am53 *cI*857 *r*95, or *r*96 prophages in *su*^+^ hosts (hence the prophages were phenotypically *N*^+^) were defective in *ori*λ replication initiation, were 7- to 17-fold reduced in *pR-Q* transcription, and did not yield any increase in phage titer after prophage induction. Five of the nine *r* mutants have now been sequenced and each has a small, in-frame deletion within *O*. In contrast, among hundreds of RK^−^ ilr mutants isolated from a defective *N*^+^ prophage (Figure 1), none were identified with in-frame deletions in *O*. Nor, have other instances involving use of the PDS, or similar selections resulted in small in-frame deletions within O being reported [12,16,17,58,59]. The major theme of those reports was that perturbing the expression of *pR-O-P* can influence replication initiation. The recent documentation on the lethal effect of *P* expression (see [25,36] and included references) may help to explain the selection differences, i.e., constitutive *P* expression is lethal to a cell, even if there is no replication initiation from *ori*λ. Rambach’s study required several other unstated assumptions: (i) replication initiation will occur from an induced *N* mutant prophage with reduced transcription of *O-P* (we note above that this was not observed for the *r*95 and *r*96 mutants); (ii) in induced *N*-defective prophage, sufficient rightward transcription occurs across (or near to) the replicator (*ori*λ) site to provide the requirement for transcriptional activation (as noted above, even when the *r*-mutant prophages were made *su*^+^ rightward transcription across *pR*-*Q* was significantly reduced); and (iii) the constitutive expression of the replication initiation proteins O and P or other de-repressed λ gene products will not be lethal to the host cell. Assumptions (i) and (ii) may still require additional study. A previous characterization of RK^−^ ilr survivor mutations from *N*^+^ prophage, revealed that all were defective in *P* or had insertions in *O* that could limit downstream *P* expression [48], suggesting that assumption (iii) is unlikely.

## 4. Materials and Methods

### 4.1. Complementation Assays and Initial Strategy for Characterizing RK^−^ Mutants

Past studies have generated hundreds of RK^−^ mutants capable of colony formation at 42 °C. Since almost all these mutants were selected prior to an ability to combine PCR with rapid DNA sequence analysis of the generated PCR fragment, the characterization of the genetic defect that permitted cell survival and growth at 42 °C required genetic analysis. This, in principle, involved complementation analysis for expression of genes *N*, *cI*, *cro*, *cII*, *O*, and *P*. Of those mutants that retained an active *imm*λ region encoding a Ts CI repressor, the cells grown at 30 °C expressed an immune response to plating by *imm*λ phage, but not to the heteroimmune phages as λ*imm*434. Shifting the cells to growth at 42 °C inactivated the Ts CI repressor and permitted the expression of *N*, *cro*, *cII*, *O*, and *P*. RK^−^ clones were inoculated into 1.5 mL tryptone broth (TB: 10 g of Bacto Tryptone, 5 g of NaCl per liter) and grown to stationary phase at 30 °C. One-tenth mL of each culture was mixed with dilutions high titer lysates *imm*λ or *imm*434 phages carrying an amber mutation in genes *N*, *O*, or *P* (none of which—at the time—were characterized by DNA sequence analysis) plus 2.5 mL of TB top agar (0.65%) agar. The mixture was poured on TB agar (1.1%) plates that were incubated at 42 °C. In the present study lysates of λ phage with *O*am mutants were freshly prepared. A single colony of *E. coli* strain 594 or these cells transformed with different versions of O plasmids were grown in LBAmp50 broth (see footnote “a” Table 3) at 30 °C overnight. Then a mixture of cells and soft agar (3 mL of warm top agar, 0.25 mL of cells and 0.25 mL of 0.01 M MgCl_2_) was poured on the top of LB plates. After agar solidification, diluted *O*am λ phage lysates were spotted on the agar, allowed to dry, the plates were incubated inverted at 30, 37, 39 and 42 °C overnight and plaque forming units were counted. The appearance of plaques at elevated plating efficiency indicated complementation for the defective gene carried on the infecting phage by the thermally induced prophage in the RK^−^ mutant cells or expressed from the plasmid. 100% plating efficiency was equated to the titer of the amber phage mutant on *E. coli* cells with a suppressor tRNA, e.g., on strain TC600 *supE*. Very low plating efficiency (<10^−4^) suggested phage-prophage marker rescue. The ability of RK^−^ cells to complement for the wild type functions expressed from *N* or *P* has always been very simple to assess. However, the interpretation of whether *O* expressed from the induced prophage was able to complement an *O*am infecting phage proved problematic.

### 4.2. DNA Sequence Analysis of λ Phage, Prophage and Plasmid Constructs

The DNA sequencing results reported herein, for each plasmid construct and phage isolate were obtained by us using methods for colony PCR, plaque PCR, and PCR amplification of cloned regions from isolated plasmid constructs, as previously described [60]. The actual sequencing results were obtained from sequencing services at the NRC National Biotechnology Institute, Saskatoon, or were submitted to Eurofins Genomics. The oligonucleotide primers employed, Table 4, were obtained from Integrated DNA Technologies, Inc. Coralville, IA, USA. In every case, at minimum, four individual representative colony, plaque or plasmids were sequenced per construct or isolate.

### 4.3. Plasmid Constructs

The *O* gene alleles were amplified from *E. coli* strains with a prophage (e.g., each of the RK^−^ mutants shown in Figure 2) or λ phage DNA, using PCR primers L-Bam-O and R-ClaI-O. The PCR fragments were cloned just downstream of promoter *pR* between the *Bam*HI and *Cla*I restriction sites in the pcIpR-(…)-timm plasmid isolated from a *dam* host strain, as drawn in Figure 3. The R-ClaI-O primer introduces an ochre stop codon at the COOH-terminal end of *O*. Primers L-Bam-P and R-ClaI-P were used for to clone gene *P*. Primers L-Bam-O and R-ClaI-P were used to clone genes *O-P*, which include the natural TGA stop codon for *O* and an ochre codon terminating *P*. The construction of plasmids pcIpR-*P*-timm, pcIpR-*O*-timm, pcIpR-*O-P*-timm, pcIpR-*O*-36*P*-timm, pcIpR-*O*-63*-P*-timm, pcIpR-*oop*#1-timm, and pcIpR-*oop*#2-timm was as reported in [25]. The plasmids oop-O and oop-O-P, Table 2, were constructed using primers L-Bam-oop#1 or L-Bam-oop#2 and R-ClaI-O to make oop-O and R-ClaI-P to make oop-O-P and the PCR fragments were cloned between the *Bam*HI and *Cla*I restriction sites in the unmethylated pcIpR-(…)-timm plasmid. Plasmid O-SPA was constructed by removing the *Bam*HI-*P*-*Asc*I fragment from pcIpR-*P*-SPA-timm [25] and inserting the fragment *Bam*HI-*O*-*Asc*I prepared using primers L-Bam-O and R-O-AscI. This construct is described in footnote “^f^” of Table 2. The DNA template used for amplifying wild type alleles of *oop-O-P* was from λ*cI*857 [25].

### 4.4. Bacterial and Phage Strains

The genotype, source and laboratory reference number for bacterial strains 594 (Pm^−^), TC600 (Pm^+^), 594 *dnaB*-grpD55 is described in Table 10 in reference [25] as are the reference phages (see also [41]). Y836, Y836 *P*:kan (Bib11t), Y836 RK^−^ ilr566a are described in Table 8 in reference [36]. Examples showing the characterization of RK^−^ mutants can be found in [11,48].

## Figures and Tables

**Table 1:** Collection of sequenced phage mutants in O and P.

Collection Isolate	Mutated Base in λ, Mutation(s), Comment, Strain Source ^a^
Phage lysates (#)	
Mutations in *O*	
λ*cI*857 *O*am905 (#1022)	38797 G to T GAG to TAG), LT; 37 AA at N-terminal of *O*
λ [Ts] *O*am29 (#51)	38914 G to T (GAG to TAG), λ induced from AC 1966 slant R473, sc1,2; 76 AA from N-terminal of *O*
λ*cI*857 *O*am317 (#52)	39166 G to T (GAG to TAG), LT; 160 AA at N-terminal of *O*
λ *O*am8 (#50)	39301 A to T (AAG to TAG), λ induced from AC 1966 slant R377, sc1,2; 205 AA at N-terminal of *O*
λ*cI*857 *O*am8 (#1025)	39301 A to T (AAG to TAG), LT
λ*cI*857 *O*am205 (#586, 630)	39570, C to G, (TAC to TAG), WD; 294 AA from N-terminal of *O*
Mutations in *P*	
λ*imm*434*cI P*am3 (#664)	39786 (CAG to TAG), WD
λ*cI*^+^*prm*116 *P*am902 (#719,722)	39894 (CAG to TAG), GG
Host ^b^ (prophage), strain #	
C600(λ[Ts] *O*am29) #1170	38914 G to T (GAG to TAG) AC 1966 slant R473 (both sc’s)
C600(λ *O*am8) #Y1169	39301 A to T (AAG to TAG), AC 1966 slant R377 (both sc’s)
C600(λ *N*am7 *cI*857[Ind] *O*am8) #Y239	39301 G to T (GAG to TAG), WD
M72 *su*^+^ (λ *N*am7 *cI*857 r95) #Y85 ^c^	12 bpΔ λ bases 39123–39134, WD
M72 *su*^+^ (λ *N*am7 *cI*857 r96) #Y84 ^c^	15 bpΔ λ bases 39139–39153, WD
M72 *su*^+^ (λ *N*am7 *cI*857 r98) #Y88	24 bpΔ λ bases 39096–39119, WD
594(λ *N*am7 *cI*857 ti12) #188	39122 C to A (ACA to AAA), WD
Aberrant designations or with additional mutations, as received (lysate or strain #)	
λ*cI*857 *O*am8 (#16)	has nonsense mutation, but WT for *cII-O-P*-40712 in *ninB*; WS
λ*imm*434 *O*am8 (#656)	has nonsense mutation, but WT for *cII*-*O-P*-40712 in *ninB*; WD
λ*cI*857 *O*am8 (#518)	no mutation in *O*; 39786 in *P*, (CAG to TAG), same as *P*am3; WS
λ*cI*857 *O*am29 (#582)	has nonsense mutation, WT for *cII*-*O-P*-40712 in *ninB*; WS
λ*cI*857 *O*am29 (#1023)	38914 G to T (GAG to TAG); 38713 T to C (TTC to TCC); LT-MMS99
λ*cI*857 *O*am125 (#1024)	39511 C to T (CAG to TAG); 39182 C to T (TCC to TTC); and 39510 A to T (CAA to CAT); LT-MMS254
594(λ*cI*857 *O*am8) #Y49, Y52	38914 G to T (GAG to TAG), really is *O*am29; WS
C600(λ *O*am125) #1171	39182 C to T (TCC to TTC); and 39510 A to T (CAA to CAT); and 39511 C to T (CAG to TAG), AC 1966 slant R573

^a^ Known strain sources: AC, A. Campbell; LT, L. Thomason; GG, G. Gussin, WD, W. Dove; WS, W. Szybalski. Furth [37] reported the original sources of the *O*am mutations as: 8, 29 and 125 from [35], 905 from P. Toothman and I. Herskowitz, 1005 from I. Herskowitz, and 205 [38]. “AA” = amino acid(s). ^b^ Host C600 is *SupE*; The Pm^−^ hosts 594 and M72 are *sup*^o^. ^c^ Transcription from *pL* and *pR*, the lack of replication arising from *ori*λ, and the absence of any increase in phage titer following prophage induction were reported for these strains in [39].

**Table 2:** Complementation of λ *O*am mutants by alleleic forms of *O* expressed from plasmids.

Host Strains and [Plasmid] # ^a^	EOP of λ Phage with *O*am Mutants ^b^		
	λ *cI*857 *O*am905 ^c^ (37 AA of *O*)	λ *cI*857 *O*am29 ^c^ (76 AA of *O*)	λ *cI*857 *O*am205 ^c^ (294 AA of *O*)
Pm^+^ *SupE* ^d^	1.0	1.0	1.0
Pm^−^ *Sup*^o^	0	0	0
Complementation by *O* variations			
O^+^ combinations			
[O] ^e^, p465	0	0.2	0.1
[O-SPA] ^f^, p472	0	1.0	0.6
[oop-O] ^g^, p677	0.05	0.1	0.1
O null mutations ^h^			
[*O-ilr208b*], p488	0	0	0
[*O-ilr223a*], p486	0	0	0
[*O-ilr541c*], p485	0	0	0
O-origin (*ori*λ) mutations ^i^			
[*O-ori:98*], p489	0	0	0
[*O-ori:95*], p491	0.2	0.3	0.2
[*O-ori:96*], p492	0.3	1.0	0.3
[*O-ori*:ti12], p493	0.3	0.1	0.3
O-P combinations ^j^			
[O-36P], p565	0	0.01	0
[O-63P], p566	0	0	0
[O-P], p569	0	0	0
[oop-O-P] ^k^ p567, p568	0	0	0
*RK*^−^ *O*^+^ *P*^−^ prophage derived from Y836 transduced into 594			
ilr 566a ^l^	0	0	0.002
*P*::kan ^m^, Bib11t	0	0	0.001

^a^ All of the complementation studies were undertaken in strains 594[pcIpR-*O*variant-timm] or with 594 cells transduced for the λ fragment mutants (ilr566a, BiB11t) from the original RK^+^ strain Y836. The host strain 594 is designated as being nonpermissive, Pm^−^ (*sup*^o^), without an amber suppressor). The precise sequences for each of the allelic forms of *O* were amplified by PCR, cloned into plasmid pcIpR-(‥)-timm, between *BamH*I and *Cla*I sites (designated by the internal brackets), and the inserted *O*-variant fragments were each verified by DNA sequence analysis. Each plasmid includes an allele of *O* positioned as is gene *cro* in WT λ, just downstream of promoter *pR* and the consensus ribosomal binding site (RBS) for *cro*. The initiation of transcription from *pR* is regulated by the Ts CI857 λ repressor encoded on the plasmid via binding to the operator site, *oR* that overlaps *pR*. For cells grown at 30 °C the CI Ts repressor remains active, binds to *oR* and blocks transcription initiation from *pR*. When the cells are shifted to 39–42 °C the CI Ts repressor transitions from partially to fully-inactive, allowing transcription initiation from *pR* and the expression of the downstream *O* allele. ^b^ The EOP value for “0” was set to <0.001. Thus, the difference between “0” and 0.1 is more than 100-fold. The efficiency of plating (EOP) was assessed at 30, 39 and 42 °C on cell lines containing plasmids. At 30 °C the results were all 0, i.e., the EOP was <0.001. In every situation, the plating results obtained at 42 °C showed an equivalent, or somewhat higher EOP than at 39 °C. All EOP data were calculated as: titer of *O*am phage on indicated host with plasmid containing *O* allele/titer of the same phage on the Pm^+^ SupE host at same temperature. The phage titer on the Pm^+^ SupE strain was set as EOP = 1.0. All values are rounded up and are relative so that standard error is not shown but represents ±10–20% of the values indicated. Each of the plating phage were sequenced throughout the *oop-O-P-ren* genes and shown to contain only the designated *O*am mutation. ^c^ Sequence designations for the *O* mutations are shown in Table 1 with mutations introducing amber stop codons: *O*am905 at 38797, *O*am29 at 38914, and *O*am205 at 39579. To have all phages include the *cI*857 mutation the lysate #1023 was used for *Oam*29 which includes a silent mutation at 38713 (Ser to Ser). The phage nonsense mutations in *O* truncate gene expression, producing polypeptides of the length shown in the heading of Table 2, each with the N-terminal end of the WT protein. ^d^ Permissive, Pm^+^, strain was TC600 *SupE*, where the efficiency for amber suppression (not complementation) was used as the baseline for full complementation. ^e^ Sequence for *O* (λ WT bases 38686–39582), representing 299 codons, plus an ochre stop codon was inserted to make plasmid pcIpR-*O*-timm. ^f^ Fusion construct represents WT *O*-(7 amino acid linker GGSGAPM)-69 amino acid SPA tag sequence-ochre stop codon. The SPA tag sequence at COOH end of *O* includes a calmodulin binding site, a TEV (tobacco etch virus) protease cleavage site and 3X FLAG sequence [47]. ^g^ Results approximate data for two plasmid constructs oop#1-*O*, representing λ WT bases 38559–39582, and oop#2-O, representing λ bases 38546–39582, each inserted between the BamHI and ClaI sites in pcIpR-(‥)-timm. ^h^ Replicative-Killing defective (RK^−^) mutants in gene *O* (see Figure 2) isolated as survivors from induced cryptic λ prophage strain Y836 as described in [11,48]. ^i^ Removed *O* fragments from replicator mutants of M72(λ *N*am7am53 *cI*857 *r95*), M72(λ *N*am7am53 *cI*857 *r96*), and M72(λ *N*am7am53 *cI*857 *r98*) lysogens from Rambach [20] via WD. ^j^ Plasmids were described in [25]. Each plasmid includes the intact sequence of *O* (λ bases 38686–39582 plus the N-terminal portions of gene P followed by ochre stop codon: 38686–39687 = O-P36, 38686–39768 = O-P63, 38686–40280 = O-P. Note that *O* and *P* are in different reading frames. ^k^ Plasmids p567 oop#1-O-P and p568 oop#2-O-P include λ bases 38559–40280 and 38546–40280, respectively, cloned between the *BamH*I and *Cla*I sites in the pcIpR-(‥)-timm plasmid [25]. The oop-O-P line represents equivalent data for plasmids oop#1-O-P and oop#2-O-P. ^l^ The RK^−^ ilr mutant 566a derived from strain Y836 was transduced using P1 with the marker *nad*57::Tn*10* that was inserted contiguous to the chromosomal λ fragment. Then the Tet^R^ *imm*λ region was transduced into strain 594. ^m^ The *kan* marker was introduced into gene *P* in strain Y836 by recombineering (=strain Bib11t) and the defective λ fragment was transduced into 594 cells as indicated for moving mutation 566a (see [25] for additional details).

**Table 3:** Does *O-P* co-expression temper P-lethality and plasmid loss?

Plasmid in 594 Host Cells	Cell Viability and (Plasmid Retention per CFU Assayed; %) at CFU Growth Temperature ^a^			
	30 °C	37 °C	39 °C	42 °C
Only *P* expression				
[P]	1.0 (35/35; 100%)	0.01 (0/35; 0%)	0.008 (0/35; 0%)	0.07 (0/35; 0%)
*dnaB*-GrpD55 [P]	1.0 (35/35; 100%)	1.0 (35/35; 100%)	1.0 (35/35; 100%)	0.98 (35/35; 100%)
Only *O* expression				
[O]	1.0 (30/30; 100%)	1.0 (30/30; 100%)	1.0 (30/30; 100%)	0.61 (29/30; 97%)
*O-P* expression combinations				
[O-P]	1.0 (62/70; 89%)	0.12 (0/70; 0%)	0.12 (0/70; 0%)	0.022 (0/36; 0%)
[oop#1-O-P]	1.0 (120/120; 100%)	0.20 (98/101; 97%)	0.005 (115/120; 96%)	0.002 (0/36; 0%)
[oop#2-O-P]	1.0 (117/120; 98%)	0.055 (76/154; 49%)	0.048 (62/120; 52%)	0.008 (0/36; 0%)
[O-36P]	1.0 (30/30; 100%)	0.79 (30/30; 100%)	0.012 (1/30; 3%)	0.0005 (0/36; 0%)
[O-63P]	1.0 (30/30; 100%)	0.90 (30/30; 100%)	0.055 (14/40; 35%)	0.0023 (0/36; 0%)

^a^ The 594 cultures with indicated plasmids were grown to stationary phase in LB (10 g Bacto-Tryptone, 10 g Bacto-Yeast Extract, 5 g NaCl per liter) plus 50 µg/mL ampicillin (=LBAmp50) for 48 h at 30 °C, diluted, spread on LB agar (includes the addition of 11 g/liter Bacto-Agar) plates (no ampicillin) that were incubated at 30, 37, 39, or 42 °C for 30 h and the average survivor titer for CFU per mL was determined for each plating temperature. Isolated survivor CFU were stabbed to LB and to LBAmp50 agar plates to estimate the proportion of CFU retaining the Amp^R^ plasmid. We tried to assay all the CFU per plate to avoid colony size discrimination, and minimally 30 CFU. The cell viability results shown in each column entry were determined by dividing the cell titer obtained for each incubation by the cell titer at 30 °C, and each value represents the average of duplicate plasmid isolates for each single experiment, with plating variations of about 10%. The results in parentheses represent the sum of results for all the CFU’s assayed from isolates. These results represent independent determinations by KR to those reported in [25], where it was shown that 594[P] cells lost 100% of their plasmids when grown at 36 °C and higher, indicating that trace levels of *P* expression (where the CI[Ts] repressor still retains some ability to block *pR*-promoted transcription below about 38–39 °C) will cure cells of the plasmid.

**Table 4:** Oligonucleotide primers employed for DNA sequence analysis and plasmid constructions.

Name	λ Map Position	Sequence (5′ to 3′) ^a^
L-37904+18	37904–37922	GCTGCTCTTGTGTTAATGG
L-MH29	37905–37922	CGTCCTCAAGCTGCTCTTGTGTTAATGG
L20	39465–39484	ACTCCGCGATAAGTGGACCC
L-22	38517–38534	TGCTGCTTGCTGTTCTTG
L-PG30	38530–38547	TTGGAACTGAGAAGACAG
L-PG1	38784–38801	AAATATGCTGCTTGAGGC
L-38985p20	38985–39005	GCAGCAAGGCGGCATGTTTGG
L-MH32	39531–39550	CACAGATCTATAGCAAACCAAAACTCGACCTGA
L-18	39980–39996	TTGCCGGAAGCGAGGCC
L-21	40360–40377	CGCAACAGTAACCAGCAT
R-PG2	40747–40764	GGTTGCGTTCCTGAATGG
L-Bam-O	38686–38718	ATATGGATCCATGACAAATACAGCAAAAATACTCAACTTCGGC
L-Bam-P	39582–39606	ATATGGATCCATGAAAAACATCGCCGCACAGATGG
L-Bam-OOP#1	38559–38580	ATATGGATCCTGGCTCGATTGGCGCGACAAGT
L-Bam-OOP#2	38546–38577	ATATGGATCCGTTGACGACGACATGGCTCGAT
L-Bam-O	38686–38718	ATATGGATCCATGACAAATACAGCAAAAATACTCAACTTCGGC
L-Bam-P	39582–39606	ATATGGATCCATGAAAAACATCGCCGCACAGATGG
R-40769m22	40747–40769	GCTGCGGTTGCGTTCCTGAATGG
R-MH33	40315–40295	GCGACGTCCCCAGGTAATGAATAATTGC
R-17	40018–40002	TAAGACTCCGCATCCGG
R-MH25	39626–39609	CTGCTCACGGTCAAAGTT
R-39280m21	39259–39280	CTGCGGCGGTCAGGTCTTCTGC
R9+1	39191–39175	TGGTCAGAGGATTCGCC
R-PG6	38569–38552	CAATCGAGCCATGTCGTC
R-1536-19	pcIpR-()-timm	GAAGACAGTCATAAGTGCGG
R-ClaI-P	40280–40259	ATATATCGATTATACACTTGCTCCTTTCAGTCCG
R-ClaI-O	39582–39559	ATATATCGATTATAGATCCACCCCGTAAATCCAGTC
R-ClaI-36P	39687–39662	ATATATCGATTACCTGCTGTACCTGCGGCTTTTCGTCG
R-ClaI-63P	39768–39746	ATATATCGATTACTTCGTTCTGGTCACGGTTAGCC
R-AscI-O	39582–39559	ATATGGCGCGCCGCTGCCGCCTAGATCCACCCCGTAAATCCAGTC

^a^ The portion of primer sequences shown in smaller font size contain restrictions sites used for cloning into plasmids and are not included within λ map sequence shown.

## Abbreviations

**Unique λ Terminology:**

**Replicative Killing (RK^+^) competent phenotype:** A property of *E. coli* cells that possess a “defective” λ prophage that is blocked by one of several means for chromosomal excision. The initiation of bi-directional replication from the prophage, which is normally prevented by an action of the prophage CI repressor—until repression is relieved, results in replication forks that move outward from the defective prophage into the *E. coli* chromosome. Massive cellular killing occurs, likely due to collisions between *E. coli*-initiated and λ-initiated opposing replication forks. **Replicative Killing (RK^−^) defective phenotype:** Mutants originating in RK^+^ cells that have lost the RK^+^ phenotype. There are numerous possibilities for **RK^−^ mutants**, but they all relate to being defective in a phage or host function required for the initiation of λ bi-directional replication. **RK^−^ Hd^−^ mutants** are defective in a host function required for λ replication initiation. **RK^+^ ilr mutants** are defective in a λ function required for the initiation of λ bi-directional replication, where ilr designates initiation of λ replication defective **RK^−^ FI^−^ mutants** represent RK^−^ mutants where the *imm*λ region of the defective prophage cannot be rescued using a phage-prophage marker rescue recombination assay. The hundreds of mutants characterized have resulted from large chromosomal deletions where the deletion endpoints straddle or partially straddle the defective prophage; but they could also represent defects in *pR*, preventing *pR-cro-cII-O-P* transcription. ***imm*λ region:** part of the λ genetic map between genes *N* and *cII* that includes the genetic elements *oL/pL-rexB-rexA-cI*[Ts857]*-oR/pR-cro*. ***imm*434 region:** the region of a λ*imm*434 hybrid phage where the *imm*λ region of λ is replaced by DNA from phage 434, but all the remaining portions of the phage genetic map are the same as for λ. **FI (functional immunity) assay:** a marker rescue assay where the *imm*λ region of a prophage (in RK^+^ cells) is rescued by an infecting λ*imm*434 phage, where the recombinant λ*imm*λ phage released can form plaques on cells lysogenized by a λ*imm*434 prophage. ***ori*λ region:** A region within λ gene *O* that contains four iteron (or ITN) sequences each containing an 18 bp inverted repeat of hyphenated symmetry (each bound by two O proteins) and an adjacent region of 39 bp termed the High-AT-RICH region that is sensitive to DNA unwinding. **PDS selection:** a selection for RK^−^ survivor mutants starting with lysogenic cells with an intact λ prophage that encodes a temperature sensitive *cI*[Ts857] repressor and is defective for *N*, such that prophage excision is prevented because genes *int-xis* are not expressed from the induced prophage, i.e., when the cells are shifted from growth at 30 °C (where the TS CI repressor is active, binds operator sites *oL* and *oR* and prevents transcription initiation from promoters *pL* and *pR*) to 42 °C where the TS CI repressor is thermally inactivated and transcription is de-repressed from promoters *pL* and *pR*. **RK^−^ selection:** same as the PDS selection except that the starting cells include an *N*^+^ cryptic λ prophage deleted for genes *int-xis-exo-bet-gam-kil* left of *imm*λ (encoding a *cI* Ts857 repressor), and all λ late genes for cell lysis and phage morphogenesis. **riC mutation:** putative new promoters arising left or right of *ori*λ that enable transcription near to *ori*λ, thus suppressing “**replicative inhibition**” caused by the loss of a *cis*-requirement for transcription from *pR* (or in the vicinity of *ori*λ), which, in addition to the activities of λ replication initiator genes *O* and *P* is a requirement for bi-directional λ replication initiation.
